# Oxidant/Antioxidant Status, PON1 and ARES Activities, Trace Element Levels, and Histological Alterations in Sheep with Cystic Echinococcosis

**Published:** 2018

**Authors:** Kıvanç IRAK, Burçak Aslan ÇELİK, Zelal KARAKOÇ, Özgür Yaşar ÇELİK, Handan MERT, Nihat MERT, Mustafa Oğuzhan KAYA

**Affiliations:** 1. Dept. of Biochemistry, Faculty of Veterinary Medicine, University of Siirt, Siirt, Turkey; 2. Dept. of Parasitology, Faculty of Veterinary Medicine, University of Siirt, Siirt, Turkey; 3. Dept. of Histology and Embryology, Faculty of Veterinary Medicine, University of Siirt, Siirt, Turkey; 4. Dept. of Internal Diseases, Faculty of Veterinary Medicine, University of Siirt, Siirt, Turkey; 5. Dept. of Biochemistry, Faculty of Veterinary Medicine, Yuzuncu Yil University, Van, Turkey

**Keywords:** Arylesterase, Cystic echinococcosis, Oxidative stress, Paraoxonase 1, Sheep, Trace elements

## Abstract

**Background:**

Total antioxidant status (TAS), total oxidant status (TOS) and oxidative stress index (OSI), nitric oxide (NO), zinc (Zn), copper (Cu) levels, paraoxonase (PON1), arylesterase (ARES) activities, and biochemical changes were studied on sheep with cystic echinococcosis.

**Methods:**

The materials were taken from 2–3 yr old sheep slaughtered in Van Province, Turkey in 2017. Before the slaughter, blood samples were collected from the healthy sheep, while various organs of animals were examined for hydatid cysts after the slaughter. Thirty sheep were protoscolex positive, hydatic group, while 30 sheep that did not have any pathological lesions in organ examinations were accepted as the control group. TOS levels, PON1 and ARES activities, and Zn levels were determined by commercial kits, while Cu levels were determined by atomic absorption spectrophotometer. The collected data were then statistically analyzed.

**Results:**

Serum TOS and OSI levels were significantly higher in sheep with cystic echinococcosis compared to the control group (*P*<0.001). TAS levels (*P*<0.01), PON1 and ARES activities, on the other hand, were significantly higher in control group compared to the cystic echinococcosis group (*P*<0.001). There were no significant differences in Zn, NO and Cu levels between the groups.

**Conclusion:**

PON1 and ARES activities increased in sheep infected with cyst hydatid. The decline of antioxidant reserves in the metabolism results in excessive amounts of free radicals, along with alterations of the normal histological structure of the cystic organ and changes in trace element metabolism.

## Introduction

The parasite *Echinococcus granulosus* causes cystic echinococcosis, which represents the settling of the larval development on the intermediate hosts. It threatens human and animal health, and it causes zoonoses. In Turkey, cystic echinococcosis is widespread due to spread of animal species it is active on, the climatic conditions, and socioeconomic development level of the society in the country. The incidence of cystic disease was reported as 0.8%–11% in different studies (1).

Eggs of *E. Granulosus* are excreted with the feces of the hosts which have the adult phase of the parasite in their intestines, and these eggs infect of various species of animals like sheep, goat, cattle, and humans, which are the natural intermediate hosts. The disease is mainly localized in the liver, but it also may spread to the kidneys, spleen, brain, bones, and heart (1, 2). The hydatid cyst cases of the hosts are difficult to clinically diagnose due to lack of strong clinical findings in most cases, and the requirement for specific parasitological examinations for differential diagnosis. Radiological diagnosis of the newly formed cysts is also difficult. On the other hand, early diagnosis is important as the success rate of the treatment increases with early diagnosis of the disease (3).

Oxidative stress plays a role in the pathogenesis of various diseases (4). In parasitic infections, the host reacts to parasites with free radicals (5) which cause oxidative stress. Control of the balance between pro-oxidants and antioxidants is crucial for the maintenance of vital and biochemical functions. Alteration of this balance in favor of pro-oxidants (oxidative stress) can lead to oxidative damage (6). Oxidative stress and antioxidant status can be assessed by several markers and various methods to measure them. However, it is both time-consuming and costly to measure these markers separately (7). For this reason, in recent years it has become more common to measure the total oxidant (TOS) and total antioxidant statuses (TAS) and to calculate the oxidative stress index (OSI) (8–10).

Cells contain antioxidant mechanisms that play an important role in protecting against reactive oxygen species by eliminating or preventing the oxidative damage (11–13). Antioxidant system is composed of antioxidant enzymes (like superoxide dismutase (SOD), catalase, glutathione peroxidase (GPx) and glucose 6-phosphate dehydrogenase (G6PD), as well as metal binding proteins (nonenzymatic substances such as transferrin, ceruloplasmin, and albumin), vitamins (alpha-tocopherol, beta-carotene) and trace elements (iron, copper and zinc) (14).

Paraoxonase 1 (PON1, EC.3.1.8.1) and Arylesterase (ARES, EC.3.1.1.2) are antioxidant enzymes recently identified (15) encoded by the same gene and have similar active centers. The enzyme PON1 has the ability to hydrolyze phenylacetate (the substrate of arylesterase) and to exhibit both arylesterase and paraoxonase activities. PON1 protects both low and high-density lipoproteins (LDL and HDL) from oxidative stress induced by free radicals (16).

Nitric oxide (NO) is a nitrogen-centered free radical that mediates both physiological and pathological events in the body and is endogenously produced by nitric oxide synthase (NOS). NO is produced in large quantities by macrophages, neutrophils and mast cells (17, 18). It contributes to regulatory effects such as anti-inflammatory, antimicrobial, and high-density conditions. NO is produced in excess in the presence of a bacterial, viral, parasitic or fungal infection in the organism (19–24).

Animals exposed to parasitic invasion undergo significant changes in blood parameters and biochemistry (25–29). Although there are various researches in biochemical parameters, there are no studies of serum TAS, TOS, OSI, NO levels, PON1, ARES, oxidant and antioxidant activities, and trace element levels in sheep infected with hydatid cyst.

In this study, we aimed to investigate the biochemical changes of cystic echinococcosis by measuring TAS, TOS, OSI, NO, Zn, Cu levels, and PON1, ARES activities in sheep sera.

## Materials and Methods

The live material of the study was 2–3-yr-old sheep brought to the slaughterhouse from controlled companies in Van, Turkey in 2017. The general health status of the sheep was checked by physical examination and blood samples were taken before the slaughter. Hydatid cyst examination was performed on various organs of animal’s post-mortem. Thirty sheep were positive (fertilized cyst) in terms of protocolex accepted as cystic group and the other 30 sheep that did not have any pathological lesions in the organ examinations and healthy in the physical examination constituted the control group. Blood samples were transported to the Siirt University Faculty of Veterinary Medicine Biochemistry laboratory and sera were removed by centrifugation at 3000 rpm for 10 min at 4 °C.

### Method

Blood TAS, TOS levels and PON1 and ARES activities were determined by an autoanalyzer (Selectra pro xl Clinical Chemistry System-The Netherlands) using commercial kits.

### Measurement of Total Antıoxıdant Status (TAS)

TAS levels were measured using commercially available kits (Relassay, Turkey). The novel automated method is based on the bleaching of characteristic color of a more stable ABTS (2,2′-Azino-bis(3-ethylbenzothiazoline-6-sulfonic acid) radical cation by antioxidants. The assay has excellent precision values, which are lower than 3%. The results were expressed as mmol Trolox equivalent/L (8).

### Measurement of Total Oxidant Status (TOS)

TOS levels were measured using commercially available kits (Relassay, Turkey). In the new method, oxidants present in the sample oxidize the ferrous ion-o-dianisidine complex to ferric ion. The oxidation reaction is enhanced by glycerol molecules abundantly present in the reaction medium. The ferric ion produces a colored complex with xylenol orange in an acidic medium. The color intensity, measured spectrophotometrically, is then related to the total amount of oxidant molecules present in the sample. The assay was calibrated with hydrogen peroxide and the results were expressed in terms of micromolar hydrogen peroxide equivalent per liter (μol H2O2 equivalent/L) (9).

### Determination of Oxidative Stress Index

The ratio of TOS to TAS is accepted as the oxidative stress index (OSI). For calculation, the obtained unit of TAS was converted to μol/L, and the OSI value was calculated according to the following Formula: OSI (arbitrary unit) = TOS (μol H2O2 equivalent/L) / TAC (μol Trolox equivalent/L) (10).

### Determination of PON1 and Arylesterase Activities

Paraoxonase 1 activity of the serum samples was investigated using the “fully automated” method developed by Rel Assay Diagnostics (Mega TNp, Gaziantep, Turkey). According to this method, paraoxonase activity is measured in medium without NaCl (basal paraoxonase activity) and with NaCl (salt-stimulated paraoxonase activity). Hydrolysis of the paraoxone (diethyl-nitrophenyl phosphate) is monitored with the follow-up of the increase of absorbance at 37-C and 412 nm. The amount of p-nitrophenol resulting from the hydrolysis is calculated using the molar absorption coefficient 17000 Mj1cmj1 (at pH 8). Net value with enzymatic activity is calculated by subtracting the basal activity value from the salt-stimulated activity value. The results are expressed as unit per liter, which is equal to the hydrolysis of 1 micromole substrate in 1 min and 1 L.

Paraoxonase arylesterase activity of the serum samples was also measured using the fully automated method developed by Rel Assay Diagnostics (Mega TNp, Gaziantep, Turkey). Phenylacetate is used as a substrate for the measurement of arylesterase activity, and phenol and acetic acid form with the hydrolysis of phenylacetate. The resulting phenol joins to 4-aminoantipyrine and potassium ferricyanide and is measured with the colorimetric method. Arylesterase enzyme activity is calculated from 4000 Mj1cmj1, which is the molar absorption coefficient of the resulting colored complex. Once again, the results are expressed as unit per liter, which is equal to the hydrolysis of 1 micromole phenylacetate in 1 min and in 1 L.

### Measurement of Nitric Oxide, Zinc and Copper Levels

Nitric Oxide (NO) levels measurement was carried out on a “Rel Assay” brand Eliza reader and Eliza washers using a commercial kit (Rel Assay Diagnostic Kits). Serum copper (Cu) and zinc (Zn) were determined by atomic absorption spectrophotometer.

### Histological Examinations

The lesional tissue samples were kept in 10% formalin solution. Following the wash, routine histological determinations were made starting from the 70% alcohol series. Serial sections with a diameter of 5 micrometers were taken from the tissues in paraffin blocks by the Rotary Microtome. Acceptable sections were evaluated with Crossman’s triple dyes. After the staining, the slides were examined under a Nikon-Eclipse 400 DSRI microscope with an attached Nikon digital camera (NIS Elements Imaging Software (ver. 3.10).

### Statistical analysis

SPSS 16.0 Windows program (SPSS Inc., Chicago, IL) was used for statistical analysis of the data. Independent t-test was used when differences between groups were determined and the results were given as mean ± SEM (Standard Error of Mean). *P*<0.05 was considered statistically significant.

## Results

Table 1 represents the obtained TAS, TOS, PON1, ARES, OSI, NO, Zn and Cu levels from the healthy sheep and the sheep with echinococcosis. Upon inspection, TAS levels for healthy and diseased animals are 1.31±0.19 and 1.21±0.03, respectively (*P*<0.01). Similarly, TOS, PON1, ARES and OSI value couples for healthy and diseased animals were also shown in Table 1. The difference between all these values was found to be statistically relevant as well (*P*<0.001). Serum Zn levels for echinococcosis group was found as 97.09±3.82, while it was 108.68±3.41 for the healthy group. The differences of NO, and Cu levels for the healthy and disease groups were not found to be statistically relevant.

**Table 1:** The obtained results for the studied parameters in healthy sheep and sheep with echinococcosis

***Parameters***	***Groups (n=30)***	***Mean± Std. Error Mean***	***P-value***
TAS (mmol trolox Equiv./L)	Cystic	1.21±0.03	.003
	Healthy	1.31±0.19	
TOS (μmol H2O2 equiv./L)	Cystic	8.47±0.50	.000
	Healthy	5.83±0.19	
OSI (%)	Cystic	0.75±0.07	.000
	Healthy	0.44±0.02	
PON1 (U/L)	Cystic	739.87±68.98	.000
	Healthy	1182.47±77.18	
ARES (U/L)	Cystic	1241.03±81.35	.000
	Healthy	1731.73±74.37	
Zinc (μg/dl)	Cystic	97.09±3.82	.027
	Healthy	108.68±3.41	
NO (μmol/L)	Cystic	5.40±0.31	.040
	Healthy	6.30±0.29	
Cu (% μg)	Cystic	112.03±3.47	.397
	Healthy	107.40±2.47	

**Fig. 1:**
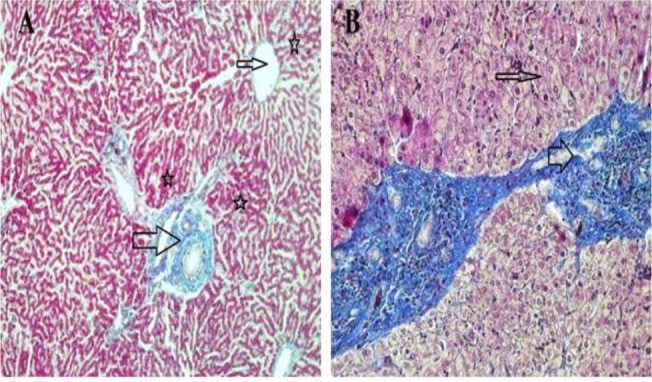
**A)** Normal liver tissue, portal area (thick arrow), vena centralis (thin arrow), hepatocytes (star), (Crossman’s triple staining 10×). **B)** Cystic liver tissue, hepatocytes (thin arrow), cystic zone (thick arrow) (Crossman’s triple staining 20×) (Original)

**Fig. 2:**
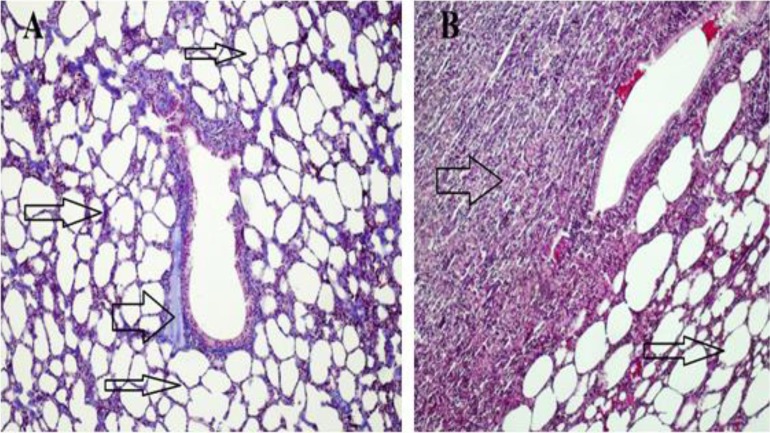
**A-** Normal lung tissue, alveoli (thin arrows), bronchial (thick arrow) containing hyaline cartilaginous part. **B-** Cystic lung tissue, normal alveolar structure (thin arrow), cystic area (thick arrow) (Crossman’s triple staining 10×) (Original)

Histological evaluation revealed that the liver and lung tissues without cysts were in normal structure (Fig. 1-A, Fig. 2-A).

**Fig. 3:**
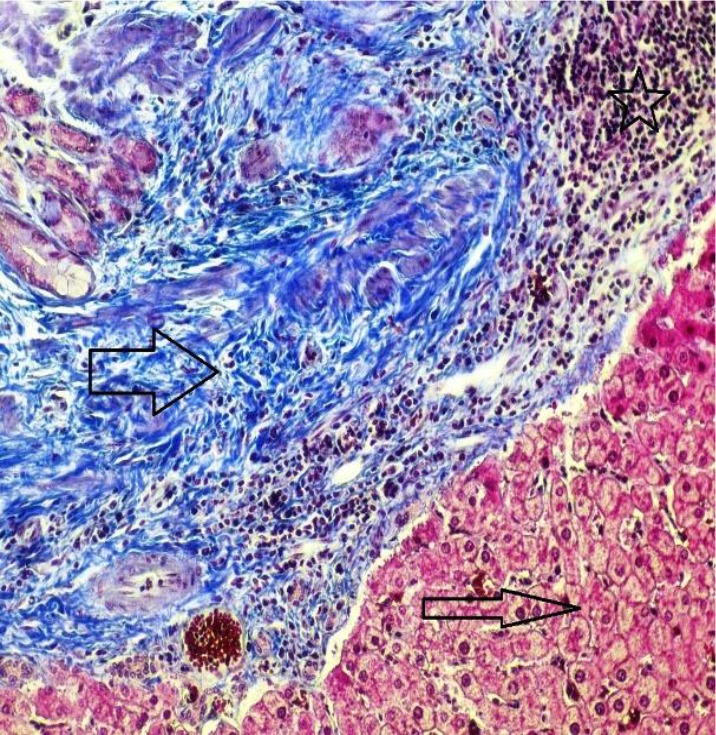
Normal liver region and hepatocytes (thin arrow), dense vascular connective tissue enlargement (thick arrow) in the cyst area, lymphocyte infiltration in the cystic area (star) (Crossman’s triple staining 20×) (Original)

**Fig. 4:**
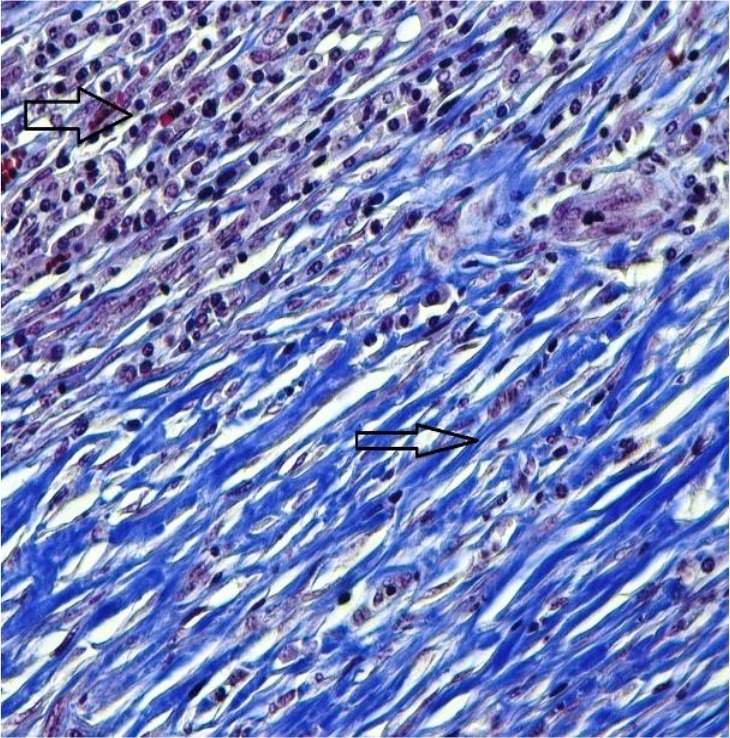
Intensive connective tissue enlargement (thin arrow) in the cystic region of the lung, mononuclear cell infiltration in the cyst area (thick arrow) (Crossman’s triple staining 40×) (Original)

Histopathologic examination of the cystic tissues revealed mononuclear cell infiltrations with increased vascolitic connective tissue (Fig. 1-B, Fig. 2-B). The cystic region of the liver displayed old hemorrhage sites filled with mononuclear cell infiltrations, mostly caused by lymphocytes. At the same time, the Remark cords were observed in a deteriorated state (Fig. 3). In the cystic region of the lung, increased connective tissue between the alveoli and mononuclear cell infiltration were noticed, which were mostly neutrophils (Fig. 4). In addition, parasites on the tissues have also been found on migration routes.

## Discussion

*E. granulosus*, a cosmopolitan parasite, is endemic in every continent. These can be seen in Central America, South America, western part of Europe, the Middle East, North Africa, Russia and China. Van and surrounding villages found that cyst hydatid disease was more prevalent especially in animals older than 2 years. In the direction of the preliminary study, it was concluded that the cystic organs were fed to the surrounding dogs and the unconsciously organs were disintegrated during the cutting and the parasite life cycle would continue and a public health problem would be evident (30).

In this study, antioxidant and oxidant parameters and mineral substances were investigated. Increased oxidant levels were observed while total antioxidant levels were decreased. Except Cu levels, changes in all parameters were statistically significant between healthy and cyst hydatid group. Histopathologically examined tissues showed mononuclear cell infiltration, lymphocyte infiltration, dense vascular connective tissue enlargement in the cystic area (Figs. 1–4).

The hydatid cyst causes phagocytic cell activation in the immunosuppressive system of the host organism, resulting in the release of reactive oxygen species and reactive nitrogen products by host macrophages and leukocytes in response to the cyst pathogenicity (31).

Although studies of oxidative stress have been reported in humans (32–34), camels (35), sheep (36) and cattle (37, 38), there has been no study in the sheep with cystic echinococcosis infections where TAS, TOS, PON1, ARES, NO levels and trace elements such as zinc and copper were studied together.

A study was conducted in which 30 cyst hydatid positive patients with Indirect Hemagglutination Test and *Echinococcus granulosus* IgG ELISA test and 35 healthy subjects whose Indirect Hemaglutination and *Echinococcus granulosus* IgG ELISA test were negative and had no parasites in the excrement in the parasitic examination (the control group) (39). Increased oxidative stress was reported in patients with hydatid cysts. The serum total antioxidant status, total oxidant status levels, and oxidative stress index levels were found to be significantly higher in hydatid cyst patients and paraoxonase and arylesterase activities in hydatid cyst patients were significantly lower compared to the values of the control group.

In another study investigating arginase activity and total oxidant/antioxidant capacity in cows with lung cystic echinococcosis, there was a significant increase in arginase activity, TOC and OSI and a significant decrease in TAC in the infected group compared to the control group (*P*<0.001) (38). Significant differences were found between groups in terms of TAS (*P*<0.01), TOS, OSI, PON1 and ARES levels (*P*<0.001) and these results are in agreement with the results reported in the literature (Table 1).

Antioxidant and nitric oxide status were investigated in patients diagnosed with *Echinococcus granulosus* reported that the level of NO was reduced in cystic echinococcosis patients compared to healthy subjects in a statistically significant manner (34). In our study, serum NO levels (5.40±0.31) of cystic echinococcosis sheep were found to be lower than those of healthy sheep (6.30±0.29). The low level at the NO level can be mainly attributed to decreased stimulation of the cell-mediated immune system.

The antioxidant system also includes iron, copper, and zinc trace elements (14). These are important in terms of the cellular damage caused by reactive oxygen species in the enzymatic defense systems. Copper, zinc, and manganese are the basic component of super-oxide dismutase (SOD) (40).

In a study of zinc, copper and iron concentrations in serum with some oxidative stress parameters in camel with cystic echinococci, serum zinc concentration was found to be significantly lower than that of healthy controls, but there was no statistically significant difference between the groups in terms of Cu level (35). When the Zn and Cu levels were evaluated in this study, Zn amounts of cystic and healthy groups were found as 97.09±3.82, 108.68±3.41, respectively. In the same manner, the amount of Cu in cystic and healthy subjects was found to be higher than those of the healthy group (112.03±3.47 against 107.40±2.47). These findings are consistent with the results reported by Heidarpour et al. (35). Changes in mineral levels were a reflection of the oxidative stresses.

## Conclusion

Paraoxonase and arylesterase activities are decreased due to increased oxidative stress in sheep infected with cyst hydatid. This can lead to tissue necrosis and increased inflammation. The decrease in antioxidant reserves results with excessive amounts of free radicals in the sheep with cystic echinococcosis, along with alterations of the normal histological structure of the cystic organ and changes in trace element metabolism.
